# Primary tumour PSMA intensity is an independent prognostic biomarker for biochemical recurrence-free survival following radical prostatectomy

**DOI:** 10.1007/s00259-022-05756-2

**Published:** 2022-03-17

**Authors:** Matthew J. Roberts, Andrew Morton, Nathan Papa, Anthony Franklin, Sheliyan Raveenthiran, William J. Yaxley, Geoffrey Coughlin, Troy Gianduzzo, Boon Kua, Louise McEwan, David Wong, Brett Delahunt, Lars Egevad, Hemamali Samaratunga, Nicholas Brown, Robert Parkinson, Louise Emmett, John W. Yaxley

**Affiliations:** 1 Department of Urology, Royal Brisbane and Women’s Hospital, Brisbane, 4006 Australia; 2 Faculty of Medicine, University of Queensland Centre for Clinical Research, Brisbane, Australia; 3 Department of Urology, Redcliffe Hospital, Brisbane, Australia; 4 Faculty of Medicine, The University of Queensland, Brisbane, Australia; 5 School of Public Health and Preventive Medicine, Monash University, Melbourne, Australia; 6 The Wesley Hospital, Brisbane, Australia; 7 I-MED Radiology, The Wesley Hospital, Brisbane, Queensland Australia; 8 Department of Pathology and Molecular Medicine, Wellington School of Medicine and Health Sciences, University of Otago, Wellington, New Zealand; 9 Department of Oncology-Pathology, Karolinska Institutet, Stockholm, Sweden; 10 Aquesta Uropathology, Toowong, Queensland Australia; 11 Faculty of Medicine, University of New South Wales, Sydney, Australia; 12 Department of Theranostics and Nuclear Medicine, St Vincent’s Hospital, Sydney, Australia

**Keywords:** Prostate-specific membrane antigen, PSMA, PET/CT, Biochemical failure, Radical prostatectomy, Gleason score

## Abstract

**Purpose:**

The prognostic value of PSMA intensity on PSMA PET/CT due to underlying biology and subsequent clinical implications is an emerging topic of interest. We sought to investigate whether primary tumour PSMA PET intensity contributes to pre- and post-operative prediction of oncological outcomes following radical prostatectomy.

**Methods:**

We performed a retrospective cohort study of 848 men who underwent all of multiparametric MRI (mpMRI), transperineal prostate biopsy, and ^68^ Ga-PSMA PET/CT prior to radical prostatectomy. PSMA intensity, quantified as maximum standard uptake value (SUVmax), and other clinical variables were considered relative to post-operative biochemical recurrence-free survival (BRFS) using Cox regression and Kaplan–Meier analysis.

**Results:**

After a median follow-up of 41 months, 219 events occurred; the estimated 3-year BRFS was 79% and the 5-year BRFS was 70%. Increasing PSMA intensity was associated with less favourable BRFS overall (Log rank *p* < 0.001), and within subgroups of Gleason score category (Log rank *p* < 0.03). PSMA intensity was significantly associated with shorter time to biochemical recurrence, after adjusting for pre-operative (HR per 5-unit SUVmax increase = 1.15) and post-operative (HR per 5-unit SUVmax increase = 1.10) parameters.

**Conclusion:**

These results in a large series of patients confirm PSMA intensity to be a novel, independent prognostic factor for BRFS.

**Supplementary Information:**

The online version contains supplementary material available at 10.1007/s00259-022-05756-2.

## Introduction

The prediction of prostate cancer treatment outcomes, including recurrence after treatment of localised disease, is currently based on various tools that incorporate clinical and pathological variables. These variables include serum prostate-specific antigen (PSA), clinical stage and histological characteristics including Gleason score (GS), the proportion of positive biopsy cores, radical prostatectomy (RP) pathological stage, and surgical margin status[1]. The incorporation of modern imaging techniques, such as multiparametric MRI (mpMRI), has improved predictive accuracy for recurrent disease, but only to approximately 70% [2] so further improvements are needed.

Modern molecular imaging techniques such as prostate-specific membrane antigen (PSMA) positron emission tomography (PET) afford superior staging accuracy for assessment of metastatic disease than conventional imaging. Incorporation of pre-operative quantitative PSMA PET parameters into prediction models with clinical variables has been shown to improve prediction of lymph node metastasis after RP and extended lymphadenectomy [3]. This model included total PSMA activity (PSMA volume * mean standard uptake value), which may not be practical for routine clinical reporting. Preliminary studies suggest that PSMA PET signal intensity of the primary tumour, expressed as maximum standard uptake value (SUVmax), may have prognostic properties, including prediction of unfavourable disease, such as higher GS and stage at RP [4].

Furthermore, PSMA intensity may provide prognostic information that is independent of traditional variables to aid in the prediction of disease recurrence [4, 5]. However, available supportive data are sourced from small retrospective series so further validation is required before being incorporated with other variables to predict disease recurrence. Therefore, the aim of this study was to investigate whether primary tumour PSMA intensity on ^68^ Ga-PSMA PET, expressed as SUVmax, contributes to pre- and post-operative prediction of biochemical recurrence-free survival (BRFS) following RP.

## Patients and methods

### Population

Following ethical approval, we retrospectively evaluated 848 men with prostate cancer who underwent mpMRI, transperineal prostate biopsy, and ^68^ Ga-PSMA PET/CT prior to RP between August 2014 and June 2018 [6].

### Imaging

Imaging protocols have been previously described [6]. mpMRI was routinely performed prior to prostate biopsy using a 3-Tesla Siemens Skyra platform (without endorectal coil). Experienced radiologists (collective experience > 15,000 prostate mpMRI scans) reported mpMRI images according to Prostate Imaging-Reporting and Data System (PI-RADS) version 1, as the inclusion period finished prior to release of PI-RADS version 2.

^68^ Ga-PSMA PET/CT was most commonly performed after prostate biopsy for staging purposes (median time interval 12 days; interquartile range [IQR] 7–21 days). Two scanners were used, including Phillips Ingenuity (Philips Healthcare, Netherlands) and later GE Discovery MI (GE Healthcare, USA). After a minimum uptake time (45–60 min) following injection of Glu-NH-CO–NH-Lys (Ahx) – HBEDCC (Esma HBED), low-dose CT scans were acquired using a 128-slice CT for anatomical localisation and attenuation correction. A contrast-enhanced diagnostic CT was also performed per institutional protocol. Detailed acquisition protocol has been published previously [7]. Images were reported by experienced nuclear physician radiologists (average > 200 ^68^ Ga-PSMA PET/CT scans/month across all prostate indications [primary staging, re-staging, metastatic disease assessment]). Intra-prostatic SUVmax values were near routinely reported, with missing values obtained via retrospective access and SUVmax determination performed on either Phillips IntelliSpace or Siemens syngo.via. Only the highest SUVmax value was recorded for this study due to the association with index lesion [4], as well as secondary lesions of lower SUVmax likely to be less oncologically relevant (lower grade, non-malignant PSMA signal intensity).

### Outcome measures

The primary outcome was BRFS following RP, defined as biochemical recurrence (BCR; serum PSA > 0.2 ng/ml and rising on two separate occasions) or commencement of salvage or adjuvant therapies (e.g. radiotherapy, androgen deprivation therapy) according to clinician discretion [8]. If the event did not occur, patients were censored at the date of last PSA test.

### Statistical analysis

The SUVmax values were analysed as a continuous variable and by quartiles (Q1 – Q4). Cox regression assessed the association between continuous SUVmax and BRFS with a post-estimation plot of the relative hazard vs SUVmax (compared to SUVmax ≤ 2.5) generated. The effect modification of biopsy grade on uptake was assessed by including an interaction term in a model and assessing if its inclusion significantly changed the model. Two further models adjusted for pre- and post-surgical parameters by entering them simultaneously with continuous SUVmax. The proportional hazards assumption was tested by examination of Schoenfeld residuals. Kaplan–Meier estimates of the survival function according to SUVmax quartile, overall and divided by Gleason score, were plotted with differences assessed by the log-rank test. A *p* value of < 0.05 was considered significant. All analyses were conducted in STATA v16.0MP (College Station, TX).

## Results

Among 848 men, the median age was 66 years (IQR 61 – 70) and PSA was 6.0 ng/ml (IQR 4.3 – 8.3). Most men had PIRADS 4/5 findings on mpMRI (86%) and were biopsy GS ≥ 3 + 4 (95%) (Table 1). RP GS was ≥ 3 + 4 in most men (98%) with pT-staging category ≥ 3 (52%) and negative margins (80%). The primary tumour was PSMA-avid (SUVmax ≥ 3) for 92% of the sample. The median SUVmax was 5.6 (IQR 3.9 – 8.6).

**Table 1:** Sample characteristics. * One missing. ** Two missing

	*N* = 848
Age, years; median (IQR)	66 (61 – 70)
PSA, ng/ml; median (IQR) **	6.0 (4.3 – 8.3)
PI-RADS; *n* (%) *	
2 3 4 5	50 (5.9) 68 (8.0) 360 (43) 369 (44)
Biopsy Gleason Score; *n* (%) *	
6 7(3 + 4) 7(4 + 3) 8 ≥ 9	40 (4.7) 292 (34) 197 (23) 76 (9.0) 242 (29)
RP Surgical margin; *n* (%)	
Negative Positive	681 (80) 167 (20)
RP Gleason Score; *n* (%)	
6 7(3 + 4) 7(4 + 3) 8 ≥ 9	13 (1.5) 312 (37) 266 (31) 27 (3.1) 230 (27)
Pathological T-stage; *n* (%)	
T2 T3a T3b/T4	406 (48) 303 (36) 139 (16)
SUVmax; median (IQR)	5.6 (3.9 – 8.6)
SUVmax quartile; *n* (%)	
Q1 (1.2 – 3.89) Q2 (3.9 – 5.59) Q3 (5.6 – 8.60) Q4 (8.61 – 53.8)	196 (23) 224 (26) 216 (25) 212 (25)

**Fig. 1:**
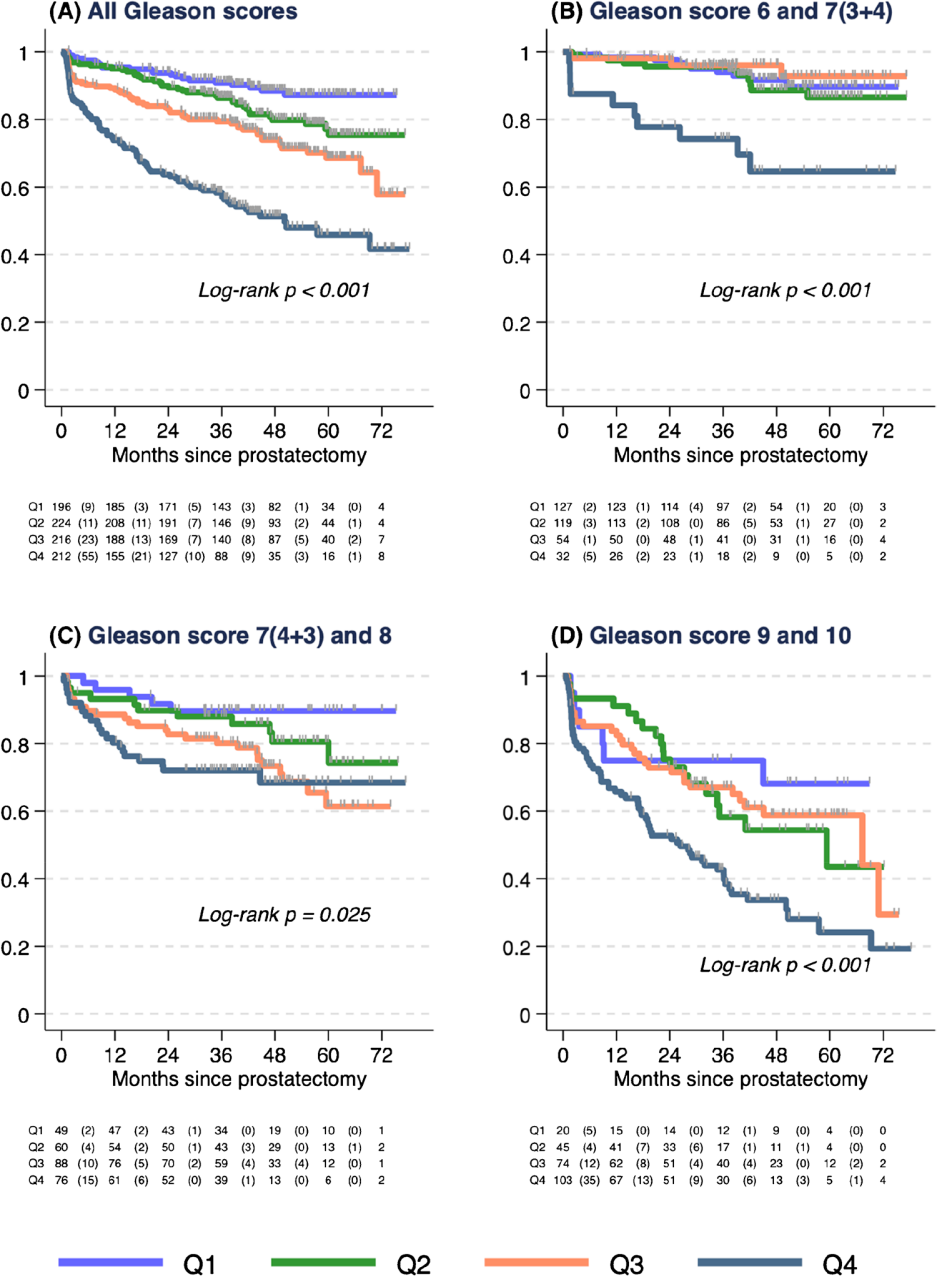
Biochemical recurrence-free survival (BRFS) after radical prostatectomy according to Kaplan–Meier survival estimate by quartiles (Q) of SUVmax (Q1 – Q4) per biopsy grade group. **A** All Gleason scores. **B** Gleason score 6 and 7(3 + 4). **C** Gleason score 7(4 + 3) and 8. **D** Gleason score ≥ 9

Median follow-up was 41 months (46 months for those without BCR or additional therapies) with the event observed in 219 men (BCR *n* = 155; additional therapies *n* = 64). The estimated 3-year BRFS was 79% (95%CI: 76–81%) and 5-year BRFS was 70% (95%CI: 66–73%). Higher Gleason score was associated with shorter BRFS (Log rank *p* < 0.001, Supplementary Fig. 1). Higher SUVmax was associated with shorter BRFS when analysed as a continuous variable (Supplementary Fig. 2) or by quartiles (Log-rank *p* < 0.001, Fig. 1A). This association remained within each Gleason score category (GS ≤ 7(3 + 4), log-rank *p* < 0.001; GS 7(4 + 3) and 8, log-rank *p* = 0.03; GS ≥ 9, log-rank *p* < 0.001; Fig. 1B–D). No significant interaction between Gleason score and SUVmax was observed (*p* > 0.9). The 3-year BRFS for SUVmax Q4 was 58% (95%CI: 51–65%), compared to SUVmax Q1 91% (95%CI: 86–94%). In the GS ≤ 3 + 4 subgroup, the corresponding estimates were 74% (95%CI: 55–86%) and 94% (95%CI: 88–97%).

In a Cox model adjusting for age and pre-surgical parameters (PSA, PI-RADS, and biopsy Gleason score), SUVmax was significantly associated with time to BRFS (HR per 5-unit increase = 1.15, 95%CI: 1.07 – 1.23) (Table 2).

**Table 2:** Cox regression with pre-surgical parameters. *N* = 844, *n* = 219 events

	HR (95% CI)
Age per 5-year increase	0.97 (0.87 – 1.08)
PSA per 5 ng/ml increase	1.15 (1.08 – 1.22)
PI-RADS	
2 3 4 5	1.0 0.80 (0.29 – 2.21) 1.02 (0.49 – 2.14) 1.85 (0.90 – 3.81)
SUVmax per 5-unit increase	1.15 (1.07 – 1.23)
Biopsy Gleason score	
6 and 7(3 + 4) 7(4 + 3) and 8 ≥ 9	1.0 2.08 (1.36 – 3.17) 5.05 (3.39 – 7.53)

In a model adding post-surgical parameters (margin status, pathological T-stage category, and RP GS), SUVmax remained significantly associated with BRFS (HR per 5-unit increase = 1.10, 95%CI: 1.02 – 1.19) (Table 3).

**Table 3:** Cox regression with pre- and post-surgical parameters. *N* = 845, *n* = 219 events

	HR (95% CI)
Age per 5-year increase	0.95 (0.85 – 1.05)
PSA per 5 ng/ml increase	1.10 (1.03 – 1.18)
PI-RADS	
2 3 4 5	1.0 0.64 (0.23 – 1.77) 0.86 (0.41 – 1.80) 0.94 (0.45 – 1.97)
SUVmax per 5-unit increase	1.10 (1.02 – 1.19)
Surgical margins	
Negative Positive	1.0 2.18 (1.62 – 2.95)
Prostatectomy Gleason score	
6 and 7(3 + 4) 7(4 + 3) and 8 ≥ 9	1.0 2.04 (1.27 – 3.25) 4.35 (2.72 – 6.96)
Pathological T-stage	
T2 T3a T3b/T4	1.0 1.51 (1.01 – 2.26) 3.31 (2.11 – 5.19)

## Discussion

Our data from 848 men confirm earlier reports that primary tumour SUVmax on ^68^ Ga-PSMA PET is a novel prognostic factor for BRFS [4, 5]. High SUVmax is associated with adverse oncologic factors, including in castrate-resistant disease with defective DNA repair alterations [9]. Furthermore, adverse clinical outcomes have been observed in patients with high PSMA expression on biopsy or RP tissue, including less favourable 5-year BRFS (26.8% vs 88.2% for low expression) and fourfold increased risk of disease recurrence [10]. Therefore, our data provide strong clinical validation that high PSMA expression indicates aggressive disease, independent of GS and other established factors.

Tools to estimate post-operative disease recurrence are limited in their accuracy [2]. Incorporation of novel imaging is a natural progression to improve predictive tools. Mazzone and colleagues showed mpMRI features in combination with biopsy GS, and PSA improved predictive accuracy compared to other classification methods [2]. Molecular tests, such as the Decipher® genomic classifier, are independently prognostic for oncological outcomes [1]; however, these tests are not commonly available outside the USA. Conversely, PSMA PET is becoming more widely available internationally. A study of 71 patients showed high PSMA intensity (SUVmax > 8) was associated with a 5.5-fold increase in hazard for BRFS in men with Gleason score ≤ 3 + 4 disease, while another cohort of 186 patients similarly found that less favourable BRFS was observed for patients with localised disease and higher SUVmax (≥ 5.4; *p* = 0.022) [5]. In combination with the findings of this study, it is apparent that assessment of PSMA intensity (via SUVmax) is a simple test that may contribute to improved prediction of survival outcomes.

Study strengths include the large sample size, high-volume imaging, and pathology providers with genitourinary expertise. Furthermore, we used the same PSMA tracer and PET machines for all patients. The findings of the study should be interpreted in the context of underlying limitations, including the retrospective review of a single-centre population. Also, SUVmax values were collected retrospectively without standardisation so may be subject to measurement and reader variation, but conversely reflect real-world outcomes. Additionally, SUVmax values are not directly transferrable due to different tracers and machines used by other centres.

In conclusion, we have confirmed with the largest cohort to date that primary tumour PSMA intensity is independently associated with post-operative BRFS outcomes. Clinicians can expect worse pathological and biochemical survival outcomes in patients with high primary tumour SUVmax values and this information may assist with patient counselling and/or repeat prostate biopsy if there is discordant pathology to imaging findings. We encourage further research to confirm these findings in relation to metastasis-free survival, as well as prospective trials using PSMA PET intensity to guide treatment decisions, such as for active surveillance of intermediate risk disease.

## Supplementary Information

Below is the link to the electronic supplementary material.

Supplementary file1 (DOCX 324 KB)

## Data Availability

The datasets generated during and/or analysed during the current study are available from the corresponding author on reasonable request.
