# Supplementary material for: Primary tumour PSMA intensity is an independent prognostic biomarker for biochemical recurrence-free survival following radical prostatectomy
Source: Eur J Nucl Med Mol Imaging. 2022 Mar 17;49(9):3289–94. doi: 10.1007/s00259-022-05756-2 (PMC9250456; doi:10.1007/s00259-022-05756-2)

**Supplementary Figure 1** – Biochemical recurrence-free survival (BRFS) after radical prostatectomy according to Kaplan-Meier survival estimate by biopsy Gleason score.


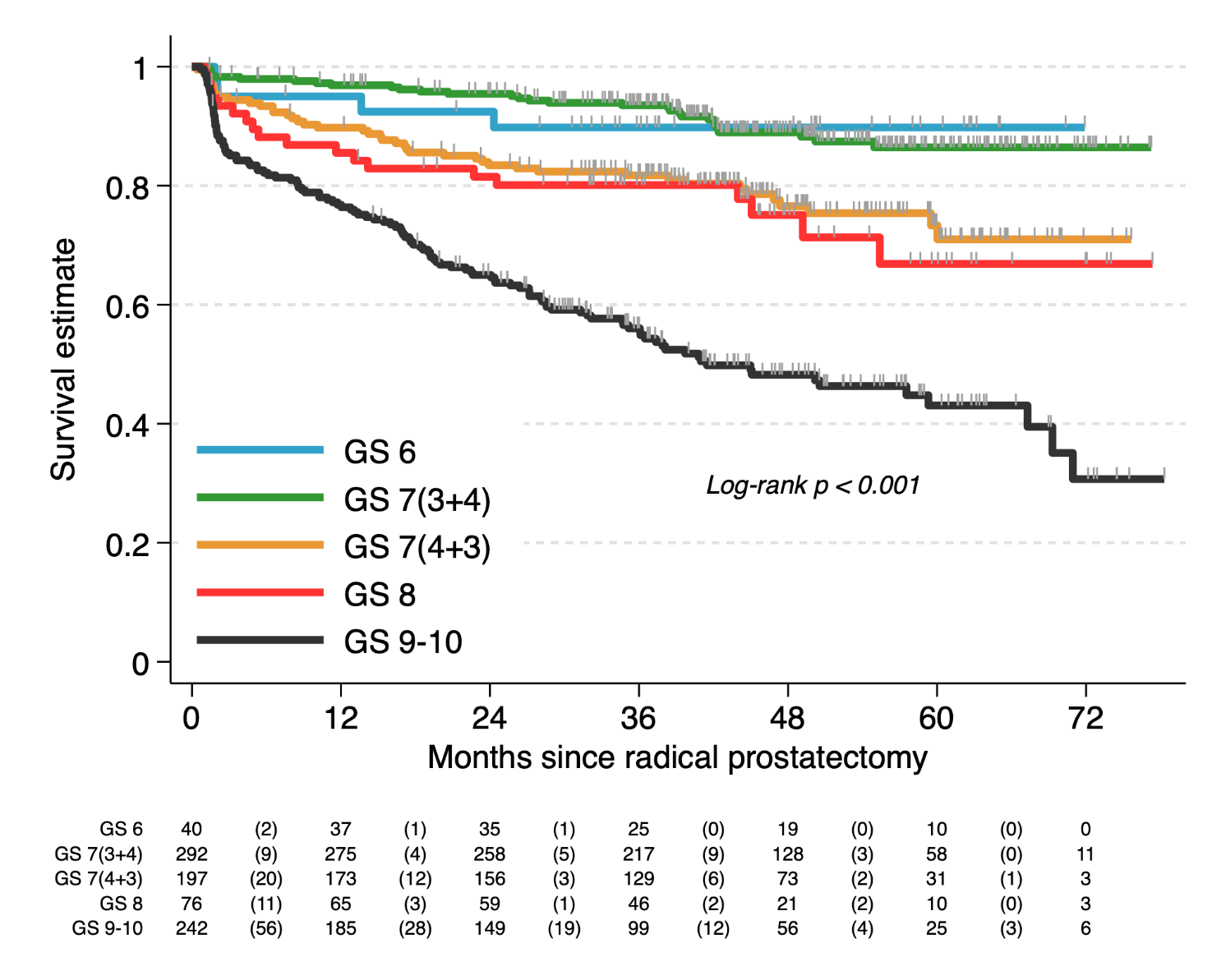


**Supplementary Figure 2** – Marginal predicted relative hazard with 95% confidence interval (grey shading) for SUVmax alone (compared to SUVmax ≤ 2.5).


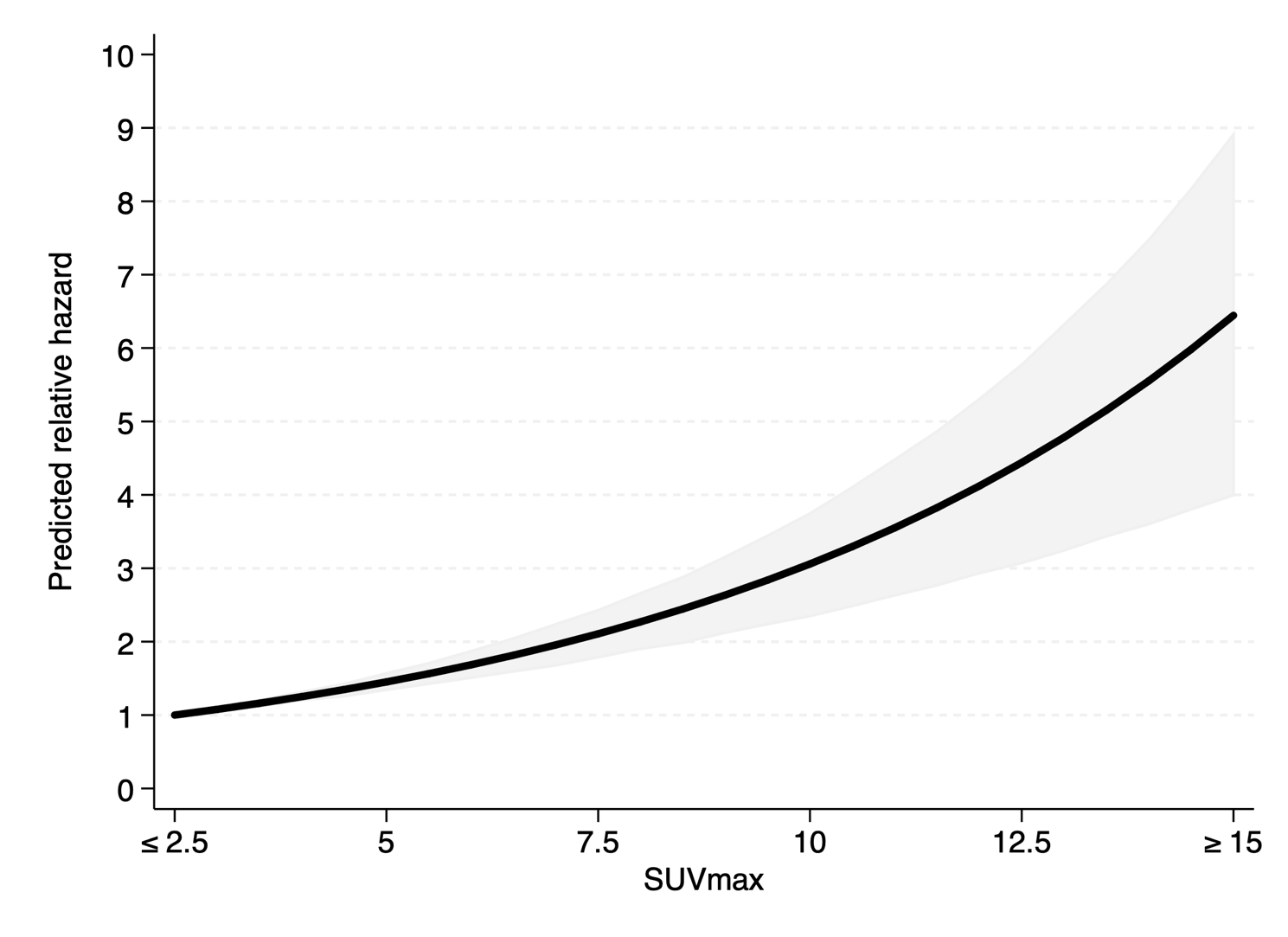

Supplement: Supplementary file 1 — Supplementary file1 (DOCX 324 KB) [file 259_2022_5756_MOESM1_ESM.docx]
